# *In Vivo* Silencing of A20 via TLR9-Mediated Targeted SiRNA Delivery Potentiates Antitumor Immune Response

**DOI:** 10.1371/journal.pone.0135444

**Published:** 2015-09-01

**Authors:** Floriane C. M. Braun, Jens van den Brandt, Sören Thomas, Sandra Lange, Juliane Schrank, Claudia Gand, Grzegorz K. Przybylski, Katrin Schmoeckel, Barbara M. Bröker, Christian A. Schmidt, Piotr Grabarczyk

**Affiliations:** 1 Clinic of Internal Medicine C, Department of Molecular Hematology, University Medicine Greifswald, Greifswald, Germany; 2 Central Core & Research Facility of Laboratory Animals, University of Greifswald, Greifswald, Germany; 3 Institute of Human Genetics, Polish Academy of Sciences, Poznan, Poland; 4 Institute of Immunology and Transfusion Medicine, University of Greifswald, Greifswald, Germany; Leiden University Medical Center, NETHERLANDS

## Abstract

A20 is an ubiquitin-editing enzyme that ensures the transient nature of inflammatory signaling pathways induced by cytokines like TNF-α and IL-1 or pathogens via Toll-like receptor (TLR) pathways. It has been identified as a negative regulator of dendritic cell (DC) maturation and attenuator of their immunostimulatory properties. *Ex vivo* A20-depleted dendritic cells showed enhanced expression of pro-inflammatory cytokines and costimulatory molecules, which resulted in hyperactivation of tumor-infiltrating T lymphocytes and inhibition of regulatory T cells. In the present study, we demonstrate that a synthetic molecule consisting of a CpG oligonucleotide TLR9 agonist linked to A20-specific siRNAs silences its expression in TLR9^+^ mouse dendritic cells *in vitro* and *in vivo*. In the B16 mouse melanoma tumor model, silencing of A20 enhances the CpG-triggered induction of NFκB activity followed by elevated expression of IL-6, TNF-α and IL-12. This leads to potentiated antitumor immune responses manifested by increased numbers of tumor-specific cytotoxic T cells, high levels of tumor cell apoptosis and delayed tumor growth. Our findings confirm the central role of A20 in controlling the immunostimulatory potency of DCs and provide a strategy for simultaneous A20 silencing and TLR activation *in vivo*.

## Introduction

A central question of cancer immunology is how tumors can evade destruction in an immunocompetent host. One of the proposed explanations is a phenomenon called immune editing where the microenvironment exerts selective pressure on the tumor cells eventually leading to malignant progression despite the presence of immune effector cells. Another possibility is the creation of an immune tolerizing microenvironment, mediated by a plethora of immune evasive or suppressive mechanisms acting in concert to counteract effective immune responses. Several of these mechanisms have been well described. These include loss of tumor antigens, alteration of antigen presentation, impaired death receptor signaling, the lack of co-stimulatory signals and the expression of tumor-derived soluble factors like cytokines or small molecules. The immunosuppressive cytokines, such as interleukin 10 (IL-10) and transforming growth factor β (TGF-β) are secreted by tumor cells and inhibit the maturation of DCs and effector T cell function through the induction of regulatory T cells (Tregs). Another crucial tumor-derived soluble factor contributing to tumor escape is vascular endothelial growth factor (VEGF), which acts as a chemoattractant for immature myeloid cells (iMC). These cells, upon the recruitment to the tumor site, develop into immature dendritic cells and macrophages, also known as myeloid-derived suppressor cells, which severely hamper T cell proliferation and eventually disable effective tumor eradication [1], [2].

The balance between activating and inhibitory signals in DCs was evidenced to play a crucial role in determining the antitumor immune response [2]. The zinc-finger protein A20 was found to be an important factor in controlling the maturation, cytokine production and immunostimulatory potential of dendritic cells [3]. A20 functions as a negative regulator of the Toll-like receptor (TLR) and tumor necrosis factor (TNF) receptor signaling pathways [3]. Specifically, it has been shown that *ex vivo* silencing of A20 in dendritic cells induced or enhanced the expression of co-stimulatory molecules, favored DC maturation and promoted the secretion of proinflammatory cytokines. In concert this resulted in significant inhibition of the Tregs and hyperactivation of cytotoxic and T helper lymphocytes. The latter produced IL-6 and TNF-α and they were refractory to Treg-mediated suppression.

A20, also called TNF-α induced protein 3 (TNFAIP3), is a negative regulator of the NF-κB pathway. With its dual ubiquitin-editing function, A20 leads to the proteasomal degradation of the receptor-interacting protein 1 (RIP1), an essential mediator of TNF receptor 1 (TNFR1) signaling complex [4], [5], [6], the TNF receptor associated factor 6 (TRAF6) [5], [7] and the I-κB kinase (IKK) [5], [8]. Moreover, A20 was shown to adjust NF-κB and MAP kinase signaling pathways as well as TNF-α-induced cell death by cooperation with the E3 ubiquitin ligases Itch and RNF11 and the adaptor proteins TAX1BP1 and ABIN-1 [4], [5], [9]. As a transcriptional target of NF-κB, A20 is a potent executor of a negative feedback loop mechanism leading to termination of NF-κB signaling [10], [11]. In the context of the immune system, A20 deficient DCs from A20^fl/fl^ CD11c-cre mice are hypersensitive to endotoxins, CpG oligonucleotides and TNF-α, and are more potent in stimulating B cells [12]. As mentioned above, *ex vivo* A20 knockdown in DCs enhanced stimulatory capacity and inhibitory effects on Tregs. This eventually shifts the balance from immune suppression to immune stimulation significantly impeding the immunotolerant tumor microenvironment.

The innate immune system gets activated by exposure to microbe associated molecular patterns (MAMPs) that are expressed by various infectious microorganisms. The recognition of MAMPs is mediated by members of the Toll-like receptor (TLR) family. Synthetic oligonucleotides (ODNs) containing CpG motifs similar to those found in bacterial DNA can efficiently induce responses similar to those observed with unmethylated CpG DNA present in bacteria. CpG ODNs are rapidly internalized by immune cells, presumably involving phosphatidylinositol 3-kinases (PI3Ks), and they interact with TLR9 that is present in endocytic vesicles. This is a highly specific interaction, since cells lacking TLR9 do not respond to CpG DNA [13]. Cellular activation triggered by the members of the TLR family, including TLR9, initiates a signaling cascade involving myeloid differentiation primary response gene 88 (MYD88), Interleukin-1 receptor-activated kinase (IRAK) and TRAF6 [11]. The cascade culminates in the activation of several transcription factors, including NF-κB, activating protein 1 (AP1), CCAAT/enhancer binding protein (CEBP) and cAMP-responsive element binding protein, which finally increases cytokine and chemokine secretion [13].

In mice, immune cells expressing TLR9 and responding to CpG stimulation belong to the myeloid lineage, including monocytes, macrophages (MΦ) and myeloid DCs [13]. Traditionally viewed as mediators of non-specific innate immune response, these cells represent the first line of host defense that limits infection shortly after exposure to pathogens [14]. In addition, innate immunity in mammals plays a pivotal role in stimulating the consecutive adaptive immune response executed by clonally expanding B and T cells [14].

In addition to its immunostimulatory properties, CpG ODNs have been used recently as carriers capable to deliver their cargo specifically to cells expressing TLR9 [15]. In an *in vitro* assay employing pooled mouse splenocytes, FITC-labeled CpG linked to siRNA were internalized by splenic DCs, MΦ, B cells, but only minimally by splenic granulocytes and T cells [15]. Upon administration of the CpG-siRNA conjugates *in vivo* the uptake of labeled CpG-siRNA was observed in resident MΦ, DCs and B cells in lymph nodes in tumor-free mice and by MΦ, DCs, myeloid cells at injection sites and in tumor draining lymph nodes (TDLN) in tumor-bearing mice [15].

The observations of the augmentation of adaptive immunity following A20 silencing as well as the dual role of CpG ODNs in targeting and stimulating TLR9-expressing DCs prompted us to develop a concept which combines these activities in a single molecule. Our concept is based on the assumption that simultaneous activation of DCs with CpG oligonucleotides and transient silencing of A20 in the same cells would act synergistically and result in significant enhancement of antitumor response (S1 Fig).

## Material and Methods

### Mice

Mouse care and experimental procedures were performed under specific pathogen free conditions in accordance with established institutional guidance and approved protocols from the animal facility of the Central Core & Research Facility of Laboratory Animals, University of Greifswald. All treatments were performed under Sevofluran anaesthesia, and all efforts were made to minimize suffering. The *in vivo* tests were performed in 8–12 weeks old female C57BL/6N healthy mice (Charles River Laboratories). The mice were divided into groups of 4 to 6 animals. The conjugates were administered by intraperitoneal injection (i.p.) of 1 nmol CpG/ CpG-siRNA constructs (against A20 or scrambled) in 200 μl. Sterile PBS of the same volume was injected into non-treated control group. To analyze cytokines levels blood samples were collected from tails every second day. At the end of experiment on day 2 to day 12 mice euthanasia was performed by CO_2_ inhalation. Whole blood was collected by heart puncture and the immune cell populations were determined. Spleen and lymph nodes were analyzed for immunophenotyping. The *in vivo* experiments in mouse tumor model were performed in C57BL/6N wild type mice (8 weeks old, female) obtained from Charles River Laboratories. The mice were divided into groups of 6 animals. For tumor treatment experiments 1x10^6 B16 melanoma cells suspended in 100μl were injected subcutaneously (s.c.) using 1 ml Micro-Fine Insulin syringes or Plastipak syringe (29G). The mice were checked daily for physical conditions, body weight, and tumor growth. On day 4 tumors reached a measurable size and their diameter was checked with a digital micrometer. The mice were treated peritumorally at two sides of the tumor with 1 or 2 nmol CpG/ CpG-siRNA constructs in 2x 5 μl using Hamilton Microliter-Syringes. As a non-treated control mice of one group were injected with sterile PBS of the same volume. Treatment was repeated daily for 7 days or until the end of experiment. Euthanasia was performed by CO_2_ inhalation when the mice developed on of the critical physical conditions (weight loss ≥ 10%, tumor size ≥ 10 mm in diameter, expression of pain, wounds, bleeding). Small amounts of blood were collected from the tail for cytokine analyses every second day. At the individual endpoint of experiment (day 10 to day 18) whole blood was collected by heart puncture to determine differences in immune cell populations. Spleen and tumor draining lymph node were dissected and the immune cell populations were analyzed. The tumor was analyzed for weight and apoptotic cells.

### Cell lines, cell culture

The murine immature DC cell line JAWSII (ATCCCRL-11904) was cultured in Complete Growth Medium (Alpha minimum essential medium with ribonucleosides, deoxyribonucleosides, 4 mM L-glutamine (Life Technologies), 1 mM sodium pyruvate (Life Technologies a Thermo Fischer Scientific Brand, Waltham, MA USA), 20% FCS, and 0.5% MycoZap Prophylactic (Lonza, Basel, Switzerland) supplemented with 5 ng/ml recombinant murine GM-CSF (PeproTech, New Jersey, USA) at a density of 1 x 10^6^ cells/ ml in 25 cm^2^ culture flasks (Greiner Bio-One, Monroe, NC, USA). The cultures were fed every two days by removing adherent cells by trypsin-EDTA 0.5% (Gibco by Life Technologies) treatment, pooling adherent and suspension cells, centrifugation (5 min, 300xg), resuspending the pelleted cells in fresh medium supplemented with GM-CSF, and placed in new culture flasks.

Bone marrow-derived DCs (preparation protocol see below) were cultured in RPMI 1640 + Glutamax (Gibco by Life Technologies) supplemented with 10% FCS, 1% MEM Non-Essential Amino Acids (NEAA, Life Technologies), 1% Sodium pyruvate (Life Technologies) and 0.5% MycoZap Prophylactic (Lonza) and 800 U/ml recombinant murine GM-CSF (PeproTech).

The murine melanoma cell line B16-F1 (Sigma-Aldrich, St. Louis, MO USA) was cultured in D-MEM (Life Technologies) supplemented with 1% Glutamine (Life Technologies), 1% MEM NEAA, 0.5% MycoZap Prophylactic, 10% FCS. The cultures were fed every two days by removing the adherent cells using trypsin-EDTA 0,5% treatment and seeding at 1:2 to 1:5 dilutions in fresh medium in new culture flasks.

### Generation of a long-term growth factor dependent murine bone marrow-derived DC (BMDC) culture

Bone marrow was isolated from femurs and tibias of C57BL/6N mice (female, 8 weeks old). The isolated bone marrow cells were suspended in RPMI, passed through a nylon mesh (70 μm Cell Strainer, BD Falcon) to remove solid tissue contaminations and red cells were lysed with RBC Lysis Solution (Qiagen, Germantown, MD USA). After washing, 1–2 x 10^6^ cells were placed in 6-well plates (Greiner CELLSTAR) in 2 ml of RPMI 1640 medium. After 1–2 hours the supernatant containing cells in suspension was discarded and the remaining adherent cells were cultured in fresh RPMI 1640 medium supplemented with 800 U/ml GM-CSF and cultured at 37°C. Macrophages were gradually depleted from the cell culture during passaging due to their stronger adherence while DCs resided preferentially in non-adherent and loosely adherent fraction of cells. After 7 days the percentage of DCs reached more than 80% (S2 Fig). BMDCs were checked constantly by fluorescence microscopy and FACS analysis. The enriched BMDCs were used for *in vitro* testing of the CpG-siRNA construct mediated A20 knockdown.

### FACS analysis

Cells were collected in phosphate buffered saline (PBS) and pre-incubated with FcR blocking reagent (Miltenyi Biotec, Bergisch Gladbach, Germany). Staining was performed in FACS buffer using fluorescence labeled antibodies. The following antibodies were used for cell characterization: CD11c (Hamster Anti-Mouse CD11c, clone HL3, BD Biosciences, San Jose, CA USA), B220 (Rat Anti-Mouse CD45R/B220, BD Biosciences), MHC II (Anti-Mouse MHC Class II, eBioscience), Ly-6G and Ly-6C (Rat Anti-Mouse Ly6G/ Ly-6C, BD Pharmingen), CD80 (Hamster Anti-Mouse CD80, Abcam), CD83 (Rat Anti-Mouse CD83, BD Pharmingen), CD86 (Rat Anti-Mouse CD86, BD Pharmingen), CD3 (Hamster Anti-Mouse CD3e, clone 145-2C11, BD Biosciences), CD4 (Rat Anti-Mouse CD4, clone RM4-5, BD Biosciences), CD8 (Rat Anti-Mouse CD8a, clone 53–6.7, BD Biosciences). The fluorescence was measured using a BD FACSCalibur flow cytometer (BD Biosciences) and data were processed with Cell Quest Pro software (Version 4.0.2 for Mac, BD Software). Forward and side scatter gating was used to restrict fluorescence analysis to intact single cells only. Analyses were performed using Win MDI 2.8.

Detection of tumor antigen TRP-1 specific CD8-positive T cells in blood mononuclear cells of tumor-bearing mice was performed using H-2Kb/TRP-1 multimer staining (TWHRYHLL Multimer; TCMetrix, Epalinges, Switzerland) and CD8 antibody (FITC Rat-Anti-Mouse CD80, BD Biosciences). For intracellular IFN-γ staining cells were fixed and permeabilized (FIX&PERM; Nordic MUbio, Susteren, Netherlands) and stained with IFN- γ antibody (PerCP/Cy5.5 anti-mouse, clone XMG1.2, Biolegend) following Multimer- and CD8-staining procedure. The fluorescence was measured with BD FACSCalibur flow cytometer and analyzed with Cell Quest Pro software.

### Cytokine quantification by CBA

For the quantification of cytokines secreted to culture medium *in vitro* and mouse serum *in vivo* the Cytometric Bead Array (CBA Mouse Enhanced Sensitivity Flex Set, BD Biosciences) was used. A panel of seven cytokines (IL-6, IL-10, IL-12p70, IL-17, MCP-1, IFN-γ, TNF-α) was measured by flow cytometry and the concentrations were quantified using the cytokine standards.

### Annexin-V-7-AAD-Staining

At the end of *in vivo* experiments, tumors were removed from sacrificed mice, cells were separated, filtered through a nylon mesh (70 μm Cell Strainer, BD Falcon), washed with sterile phosphate-buffered saline (PBS), suspended in 50 μl Annexin V binding buffer (BD Biosciences) containing 2.5 μL of Annexin V-APC and 2.5 μl of 7-AAD, and incubated for 15 minutes at room temperature in the dark. The fluorescence was measured using a BD FACSCalibur flow cytometer and analyzed with Cell Quest Pro software.

### Confocal microscopy

For confocal microscopy, cells were cultured on glass bottom dishes (WillCo-Dish GWSt-5040), fixed using 2% paraformaldehyde, washed, and mounted in Hank's balanced salt solution. Internalization of FITC-labeled CpG-siRNA constructs was analyzed by confocal microscopy using Leica TCS SP5 II Confocal Laser Scanning Microscope with HCX PL APO 63X 1.2 N/A water immersion objective and Olympus Fluoview FV1000 LSM with UPLSAPO 60X 1.2 N/A water immersion objective. The post-acquisition analysis was performed using FV1200 ASW and ImageJ 1.47v software. Cellular staining: Nucleus–Nuc Blue Live Cell Stain (ex 405), Membrane–Oregon Green 488 DHPE (ex 448), CpG-siRNA constructs–FITC (ex 494).

### RNA extraction and quantitative RT-PCR

TRIzol Reagent (Invitrogen by Life Technologies a Thermo Fischer Scientific Brand) was used for total RNA extraction. cDNA synthesis was performed using SuperScript II RT (Invitrogen). mRNA expression of A20, IκBa, IL-6, TNF-α was analyzed by real-time quantitative PCR with Platinum SYBR Green qPCR SuperMix (Invitrogen by Life Technologies) using the 7500 Real-time PCR System (Applied Biosystems by Life Technologies, CA USA). The obtained data were calculated by ΔΔCT method. ß2MG was used as a reference gene for normalization. Primer sequences are provided in S1 Materials and Methods.

### Western Blot analysis

Cells were lysed in RIPA lysis buffer (Santa Cruz Biotechnology, Dallas, Texas USA). Equal protein amounts (25 μg per lane) were separated by SDS-PAGE and equal protein transfer was controlled by Ponceau staining (not shown). A20 protein levels were determined using rabbit monoclonal antibody (D13H3; Cell Signaling Technology, Danvers, MA USA) diluted 1:1000 followed by a goat anti-rabbit-AP secondary antibody (1:3000). To ensure equal protein loading, actin levels were determined using rabbit polyclonal antibody (A2103; Sigma-Aldrich) diluted 1:1000 followed by goat anti-rabbit-AP secondary antibody diluted 1:3000. The detection was performed with the Western-SuperStar Immunodetection System (Applied Biosystems, Carlsbad, CA USA).

### Oligonucleotide design

Two different siRNAs for A20 knockdown were used in this study: siA20_5 and siA20_6. To generate mouse-specific CpG-siRNA constructs, the CpG1668 (B-type CpG) was linked to antisense (AS) strands of siRNAs using a C3 carbon chain (CH_2_)3. The resulting constructs were hybridized to complementary siRNA sense (SS) strands to generate CpG-siRNA conjugates (deoxyribonucleotides are underlined and “s” indicates phosphorothioation sites):

CpG (5’ TsCsCsAsTsgsAsCsgsTsTsCsCsTsgsAsTsgsCsT 3’), CpG-siA20_5 (antisense strand: 5’ TsCsCsAsTsgsAsCsgsTsTsCsCsTsgsAsTsgsCsT-[3(CH2)]-UUgAggCUACCUgUgUAgUUCgAgg 3’ and sense strand: 5’ CCUCgAACUACACAggTUggTUAgCCUCAA 3’), CpG-siA20_6 (antisense strand: 5’ TsCsCsAsTsgsAsCsgsTsTsCsCsTsgsAsTsgsCsT-[3(CH2)]-UCAAACAUggugCUUCCgAgUgUgC 3’ and sense strand: 5’ gCACACUCggAAgCACCAUgTUTgA 3’), CpG-scrambled siRNA (antisense strand: 5’ TsCsCsAsTsgsAsCsgsTsTsCsCsTsgsAsTsgsCsT-[3(CH2)]-gACgCACCAgCCgUUCCAAUAUACA 3’ and sense strand: 5’ UgUAUAUUggAACggCUggUgCgUC 3’)

For cellular uptake studies, CpG-siRNAs were labeled with fluoresceinisothiocyanate (FITC). Oligonucleotides were synthesized and annealed by the provider (BioSynthesis, Lewisville, TX USA). The quality was validated by MALDI-TOF mass spectrometry, HPLC and/ or PAGE, the quantity was measured twice by UV spectrophotometry.

### Ethics statement

This study was performed in strict accordance with the German Animal Protection Act (Tierschutzgesetz, 18^th^ of May 2006, Germany, in agreement with the European Directive 2010/63/EU). The protocol of the study was approved by the Animal Welfare/Ethics Officer of the University of Greifswald (Prof. Dr. Olaf Grisk) and the Veterinary and Food Control Office, State Department of Agriculture, Food Safety and Fisheries Mecklenburg-Vorpommern, Germany (Permit Number: 7221.3–1.1-063/12 “New approach in cancer therapy: induction of an antitumor immune response by CpG-siA20 treatment in the mouse”*)*. All treatments were performed under Sevofluran anaesthesia and all efforts were made to minimize suffering.

### Statistical analysis

For RT-PCR and CBA data analyses mean values of technical replicates (measured three times in duplicate) and mean values of biological replicates (number of mice per group) were used, respectively. In addition to the experiment kinetics, the mean values of all time points were integrated by calculating the area under curves (AUC) defined by individual time points. Statistical analyses were performed by applying the Kruskal–Wallis one-way analysis of variance by ranks (non-parametric method) and the Wilcoxon rank-sum test (also called Mann-Whitney U test) using SAS 9.3 software (Institute for Biometry and Informatics, University Greifswald); n.s. indicates not significant; *** indicates p<0.001; ** indicates p<0.01; and * indicates p<0.05.

## Results

### TLR9-mediated siRNA delivery into murine dendritic cells *in vitro*

Recently, a number of reports have been published which showed the stimulatory effects of A20 depletion on NF-κB in the context of immune cells. Depending on the affected cell type, A20 loss in mice can lead to signs of autoimmunity similar to human diseases like arthritis, psoriasis, and colitis depending on the affected cell type [16], [17], [18]. These observations indicate a role of A20 as a guardian of self-tolerance. However, the mechanisms of self-tolerance, being crucial to prevent autoimmunological pathologies on the one hand, are the major reasons of tumor immune escape on the other. This was evidenced by several *in vitro* and *ex vivo* studies in mice as well as in humans which showed that A20 down-regulation in DCs enhances their T cell stimulatory capacity [19] and overcomes the Treg cell-mediated suppression of anti-tumor immune effector mechanisms [20].

Inspired by the advantageous effects of A20 depletion on anti-tumor immune activity and being aware of the risk of autoimmunity, we aimed at the development of an A20-inhibition strategy that would limit the side effects as well as the need of any *ex vivo* DC manipulation. To achieve this, we adopted the method described by Kortylewski *et al*. [15],. Briefly, we conjugated double stranded, A20-specific pre-validated siRNA oligonucleotides with a TLR9 agonist: a single stranded DNA oligonucleotide encoding CpG motifs to generate CpG-siA20 constructs (S1 Fig). In addition to the stimulatory properties the CpG-ODN mediates cellular internalization of the construct followed by docking to TLR9 receptor. Targeting this intracellular receptor that is expressed mainly by DCs and macrophages allows cell-specific CpG-siRNA uptake and A20 gene knockdown *in vivo*.

**Fig 1:**
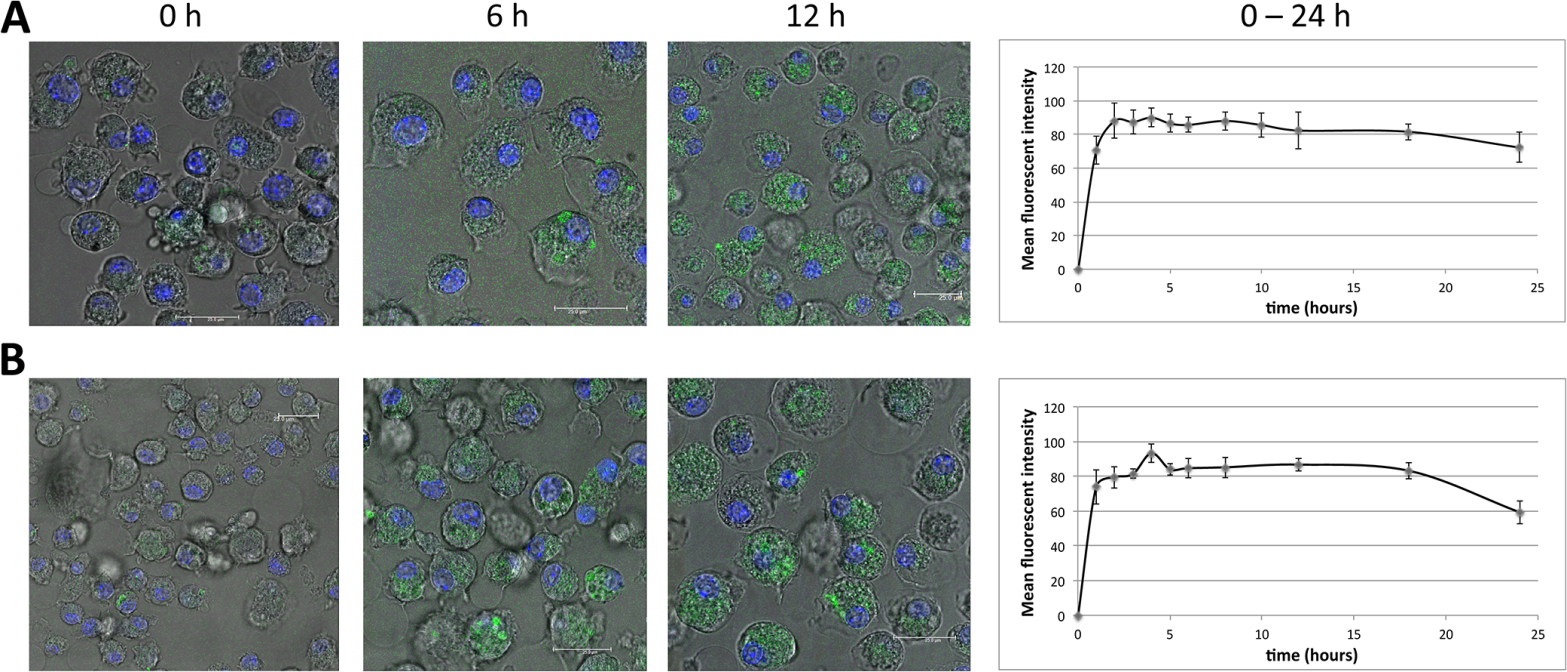
Cellular uptake of CpG-siRNA-FITC constructs by BMDCs. BMDCs on day 7 of culturing incubated with CpG-siRNA constructs linked to FITC. Confocal images of Nuc Blue (ex 405) stained CpG-siRNA A20-FITC **(A)** and CpG-siRNA scrambled-FITC **(B)** treated BMDCs from 0, 6, 12 hours and mean fluorescent intensity of FITC in 10 cells/time point over 24 hours.

**Fig 2:**
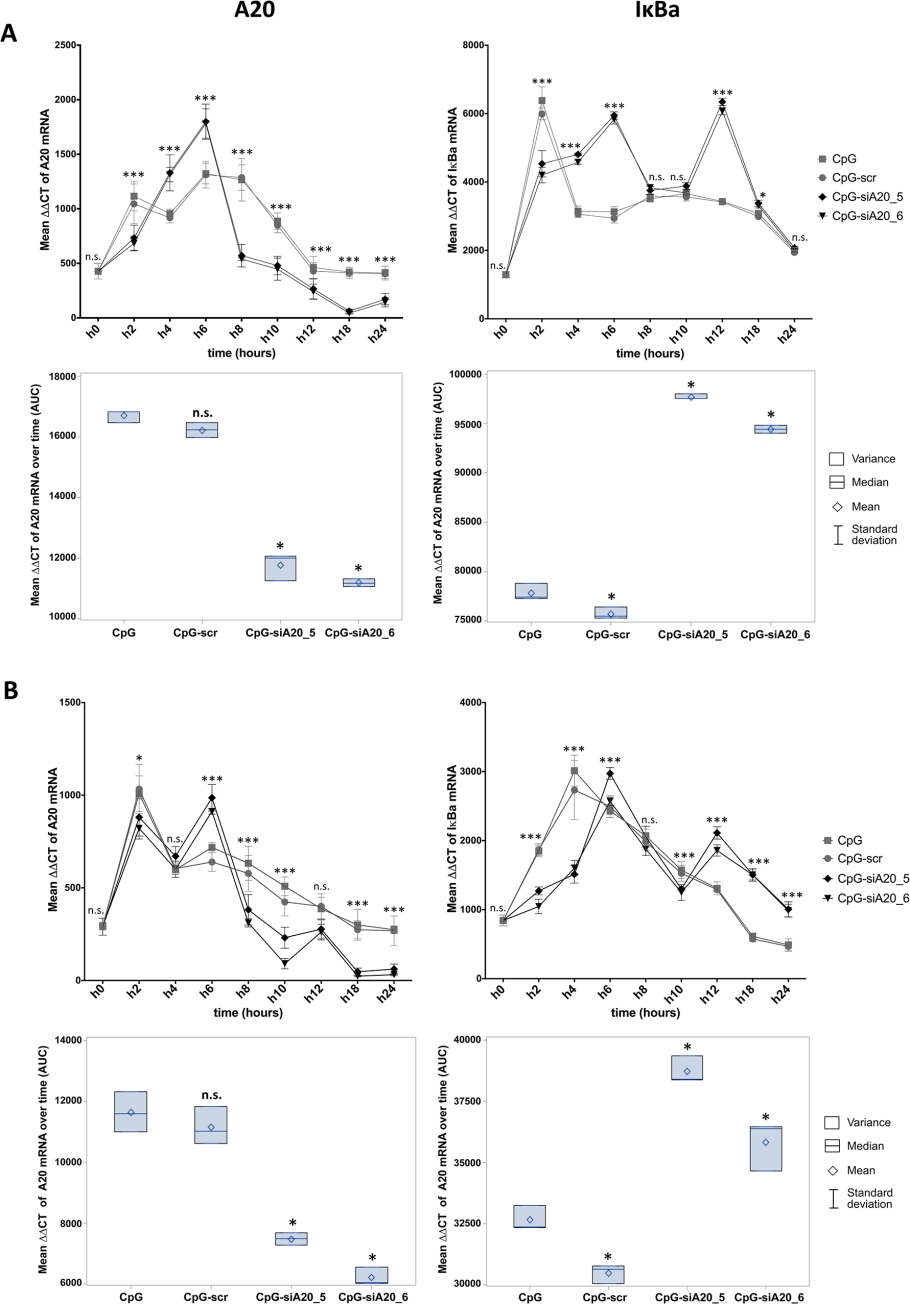
Expression of A20 and IκBa mRNA in CpG/ CpG-construct treated BMDCs and JAWSII cells. A20 and IκBa mean expression0020measured at 0–24h by qRT-PCR (∆∆CT normalized to β-2-microglobulin) in BMDCs **(A)** and JAWSII dendritic cell line **(B)**; Lower panels show integrated results of all time points calculated as areas under curves (AUC) defined by 9 time points; The results from three technical replicates of one representative experiment are shown; n.s. indicates no significant differences, * indicates p<0.05, ** indicates p<0.01, *** indicates p<0.001 compared to CpG treated cells.

**Fig 3:**
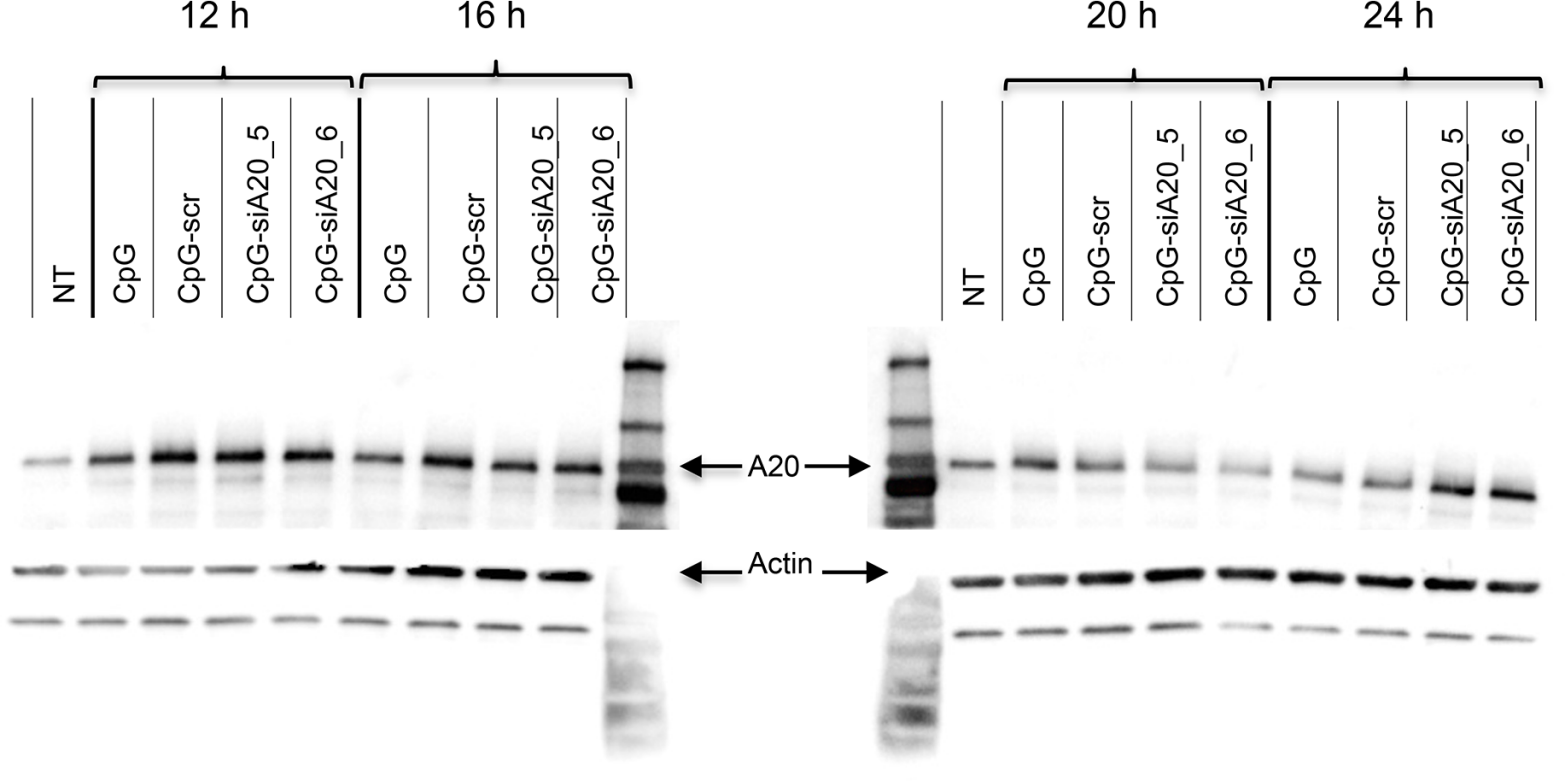
A20 protein expression in CpG/ CpG-siRNA_A20 treated BMDCs. BMDCs on day 15 of culturing were treated with 1 nmol CpG/ CpG-scrambled siRNA/ CpG-siRNA A20 constructs (CpG-siA20_5, CpG-siA20_6). Protein lysates were collected 12, 16, 20, and 24 hours after treatment and analyzed by Western Blot using A20-specific antibody and actin as loading control.

First, using confocal microscopy and fluorescence labeled CpG-siA20 constructs, we confirmed cellular uptake in BMDCs *in vitro*. Already 1 hour after FITC–labeled constructs were added to the cell culture the strong fluorescent signals were detected inside DCs (Fig 1). The efficiency of the uptake reached its peak (90% of FITC-positive cells) after 2h of incubation and approximately 80% of cells retained the FITC signal for 24 h. The uptake efficiency was equally efficient for A20-specific (Fig 1A) and scrambled control (Fig 1B) CpG-siRNA conjugates. Compared to cells incubated with control oligonucleotides (CpG alone or CpG linked to a scrambled control siRNA), the CpG-siA20-treated BMDCs expressed A20 mRNA at initially higher levels followed by marked decrease at later time points, as determined by quantitative real-time PCR (Fig 2A). This was specific for the siRNA directed at A20, since a construct containing a scrambled siRNA sequence (CpG-scr) did not have this effect. Similar results were obtained in JAWSII dendritic cell line (Fig 2B). The elevated expression of a NF-κB target, the IκBα gene in A20-depleted cells indicated enhanced activation of the canonical NF-κB pathway and confirmed the functionality of the constructs (Fig 2A and 2B). When analyzed on protein level, A20 was initially upregulated in all samples treated with CpG or with CpG-siRNA constructs irrespective of their specificity compared to non-treated control DCs. At later time points the amount of A20 protein decreased in cells with A20-specific siRNA fused to CpG. Four hours later the A20 protein signal was again elevated above the levels observed in control cells and CpG-treated cells. Most likely this displays the consequence of the prolonged and augmented NF-κB activity due to the A20 knockdown. (Fig 3)

### Immunostimulatory properties of CpG-A20 siRNA *in vitro* and *in vivo*

**Fig 4:**
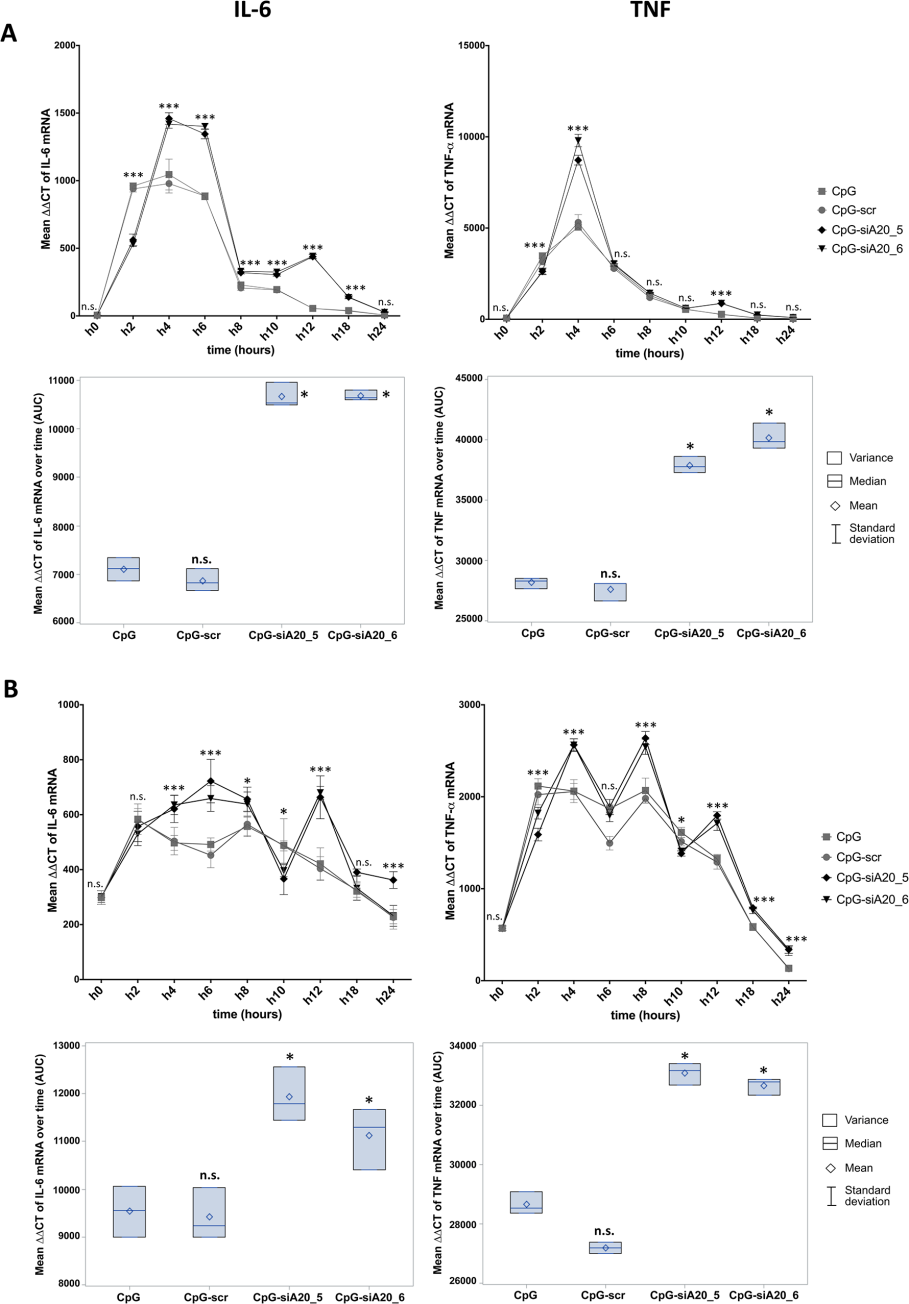
Expression of IL-6 and TNF mRNA in CpG/ CpG-construct treated BMDCs and JAWSII cells. IL-6 and TNF mean expression measured at 0–24h by qRT-PCR (∆∆CT normalized to β-2-microglobulin) in BMDCs **(A)** and JAWSII dendritic cell line **(B)**; Lower panels show integrated results of all time points calculated as areas under curves (AUC) defined by 9 time points; The results from three technical replicates of one representative experiment are shown; n.s. indicates no significant differences, * indicates p<0.05, ** indicates p<0.01, *** indicates p<0.001 compared to CpG treated cells.

**Fig 5:**
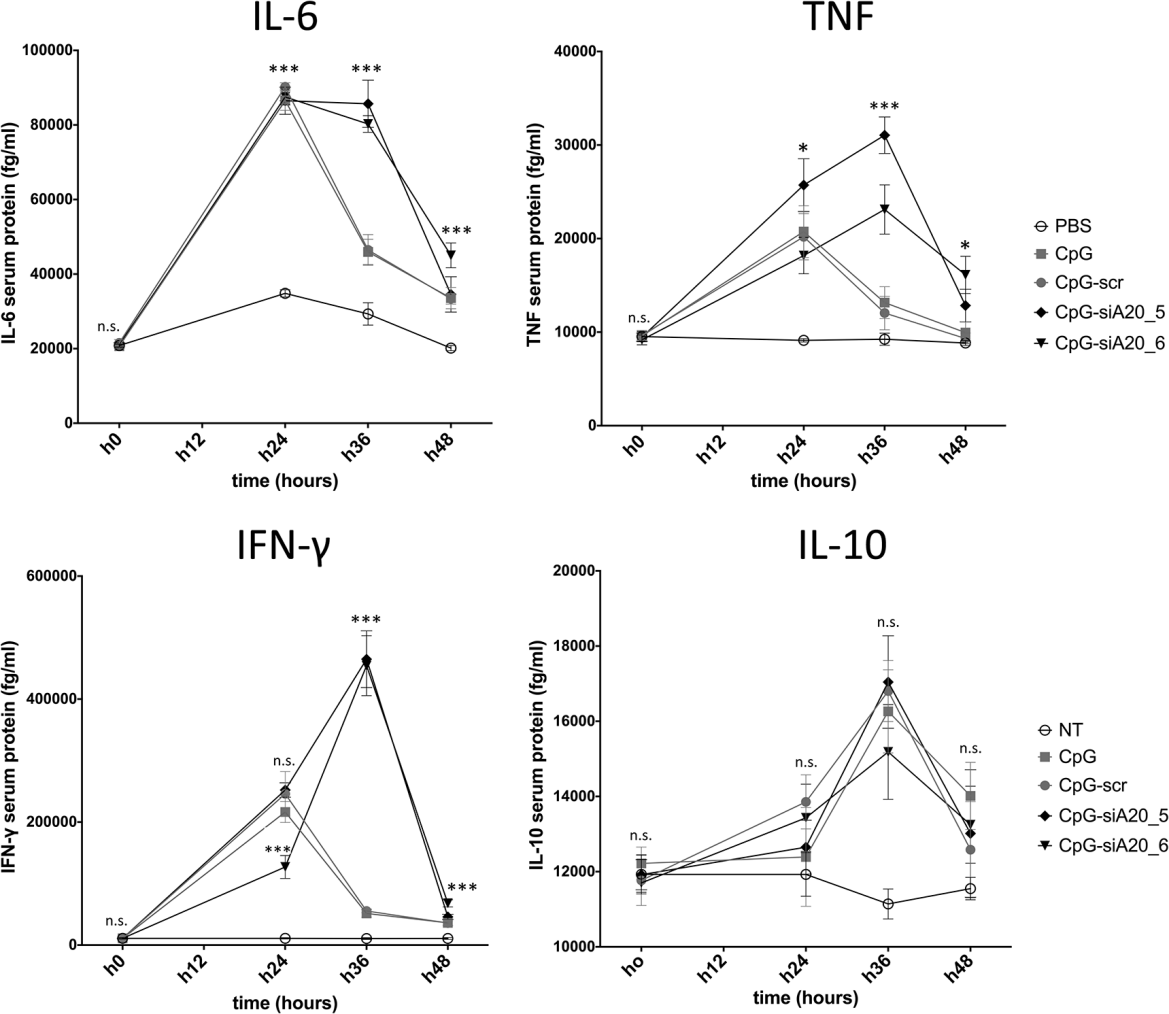
Cytokine expression in mice after intraperitoneal CpG/ CpG-siRNA treatment. Expression of IFN-γ, TNF-α, IL-6, and IL-10 after intraperitoneal injection of 1 nmol CpG, CpG-scrambled RNA or CpG-siRNA A20 in healthy C57BL/6 mice. Cytokines serum levels in pg/ml analyzed by CBA at 4 time points (0, 24, 36, 48h). Shown are representative results from one out of three experiments; n.s. indicates no significant differences, * indicates p<0.05, ** indicates p<0.01, *** indicates p<0.001.

In order to assess the consequences of CpG-siRNA-mediated A20 knockdown on DC function, we analyzed the mRNA levels of the pro-inflammatory cytokines IL-6 and TNF-α in BMDCs and JAWSII cells that were left untreated or incubated with CpG only or CpG-conjugates. Both in murine BMDCs (Fig 4A) and the immature murine DC line JAWSII (Fig 4B) the stronger induction of IL-6 and TNF-α expression was observed in CpG-siA20 treated cells. In BMDCs the induction of both cytokines reached its peak between 2–6h and was slightly higher than in JAWSII. Next, we verified the activity of the CpG-siA20 construct *in vivo*. Naïve healthy mice were treated with PBS or 1 nmol of CpG or CpG-siRNA constructs, respectively. After intraperitoneal injection, the serum levels of pro-inflammatory cytokines were measured at four time points up to 48 hours using the cytokine beads assay (CBA). As expected from former CpG studies, the assay revealed significantly higher concentrations of IL-6, TNF-α, but also IFN-γ and IL-10 in serum isolated from animals treated with CpG or with CpG-siRNA constructs compared to PBS-injected mice. Notably, in case of IL-6, TNF-α and IFN-γ the cytokine concentrations induced by the CpG-siA20 constructs were substantially higher than those triggered by treatment with CpG alone or with the CpG-scr construct indicating an A20-specific effect (Fig 5).

**Fig 6:**
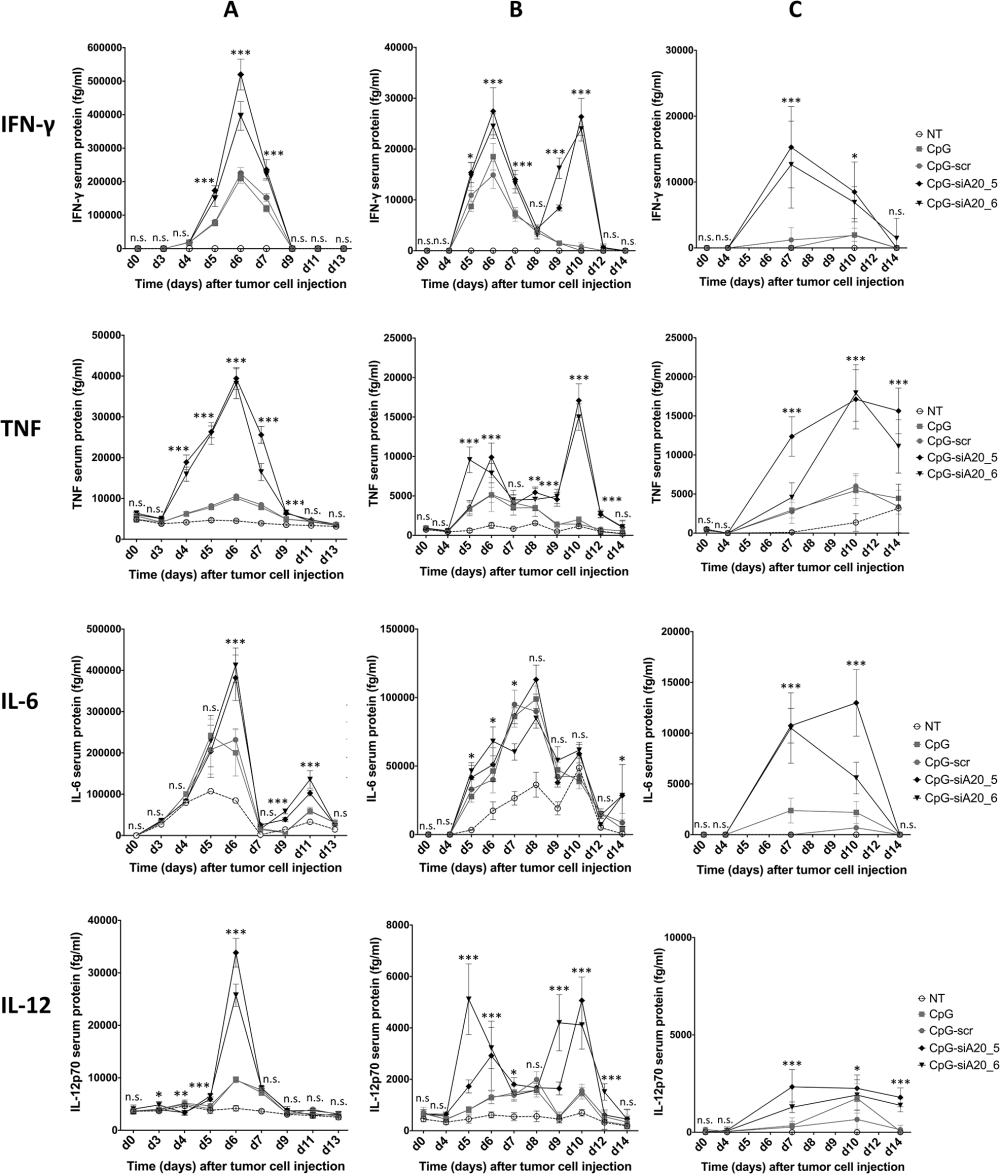
Cytokine expression in B16 melanoma-bearing mice after CpG/ CpG-construct treatment. Expression of IL-6, TNF, IFN-γ and IL-12p70 in tumor bearing mice treated daily with 1 nmol **(A,B)** or 2 nmol **(C)** CpG/CpG-siRNA conjugates 7 **(A)** or 4 days **(B,C)** after tumor challenge. Mean cytokines serum levels in fg/ml measured by CBA; * indicates p<0.05, ** indicates p<0.01, *** indicates p<0.001 compared to CpG treated cells.

**Fig 7:**
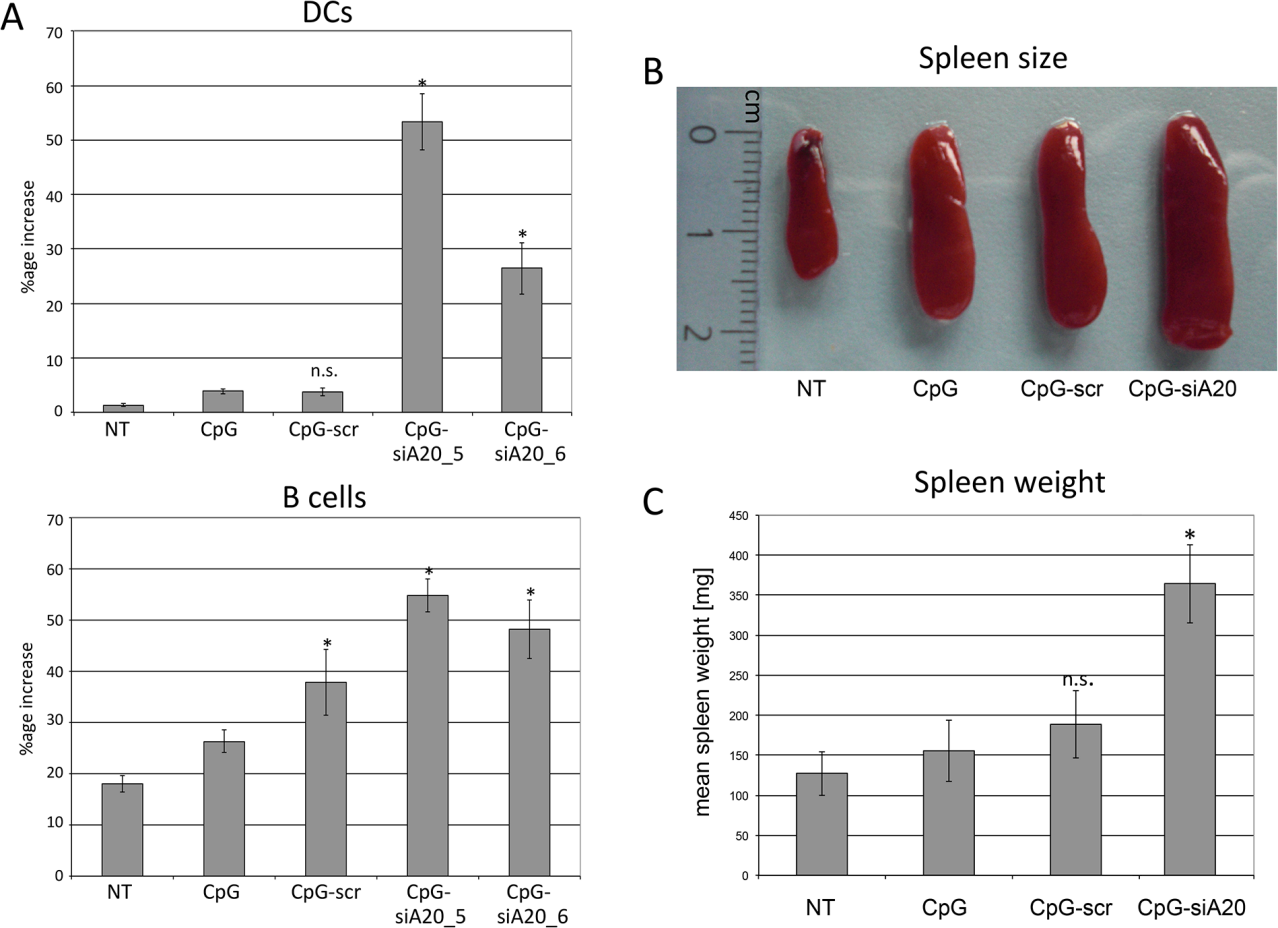
Cellular changes in blood and spleen size in mice after CpG/ CpG-construct treatment. **(A)** DC and B cell numbers in blood collected from B16 melanoma-bearing mice treated daily with CpG/ CpG-scrambled RNA/ CpG-siRNA A20 starting on day 4 after tumor cell challenge repeated daily until day 10; blood collected for cellular analyses on day 10. Shown are mean percentage increases of DCs (CD11c+ B220+) and B cells (CD11c- B220+) of 6 mice/group; n.s. indicates not significant, *indicates p<0.05 compared to CpG. **(B)** Spleens from B16 melanoma bearing mice at the end of experiment. The picture shows one representative spleen per group. **(C)** Mean spleen weight (in mg).

We next evaluated the immunomodulatory and antitumor properties of CpG-siA20 in a mouse melanoma model. First, mice were inoculated subcutaneously with 1 x 10^6^ B16 melanoma cells. Seven days later, PBS or 1 nmol of CpG or CpG-conjugates were injected peritumorally and the treatment was repeated at 24 h intervals for 7 days. Blood samples were collected every 48h starting from the first treatment and the serum levels of the pro-inflammatory cytokines were measured by CBA. Similar to naïve mice, tumor-bearing animals, which received the CpG or CpG-siRNA constructs elaborated higher serum levels of IL-6, TNF-α, IFN-γ and IL-12p70 compared to PBS-treated controls. Notably, the CpG-siA20 conjugates increased the concentration of TNF-α, IFN-γ and IL-12p70 stronger compared to CpG alone or the control CpG-scr construct. IL-6, in contrast was induced to a similar degree by all preparations containing CpG (Fig 6A). Interestingly, the peak expression of IL-6 and TNFα preceded the increased IFNγ and IL-12p70 levels suggesting the primary role of IL-6 and TNF in the initiation of the immune response in our study. To address the effects of tumor size at beginning of treatment on the efficiency of CpG-siA20 ODNs, an experiment was performed in which the treatment was initiated 4 days after inoculation of B16 cells. The CBA assay confirmed the findings of the former trial, however in this case the CpG-siA20 conjugate increased the expression of all 4 cytokines, including IL-6 above the concentrations triggered by treatment of CpG alone or the control construct (Fig 6B). Interestingly, in this experimental setting the induction of IFNγ and IL12p70 occurred in two waves separated by a drop on day 7 and 8. This observation may reflect the fluctuations of A20 protein observed earlier after CpG-siA20 treatment of DCs *in vitro* and being the consequence of NFκB activity on A20 promoter. The results obtained from the experiment with doubled CpG/CpG-siRNA amount further confirmed significant upregulation of all four analyzed cytokines (Fig 6C). To sum up, regardless of the treatment schedule and CpG or conjugates amount injected into the tumors, the A20 knockdown caused markedly higher induction of immunostimulating cytokines than CpG alone or control conjugate. In addition to the elevated cytokine serum levels, highly increased numbers of DCs were observed in the blood of CpG-siA20-treated animals. The percentage of circulating B cells was raised as well, interestingly also in animals which were injected with CpG fused to scrambled siRNA (Fig 7A). Another parameter indicating an enhanced immune response in A20-depleted animals was their enlarged spleen. Splenomegaly was recognized in all animals treated with CpG preparations but spleens isolated from CpG-siA20 animals were significantly bigger than those of controls (Fig 7B and 7C). Despite differences in size, the cellular composition of spleens was similar in all animal groups regardless of the organ size (data not shown).

### Antitumor effects of CpG-A20 siRNA conjugates

**Fig 8:**
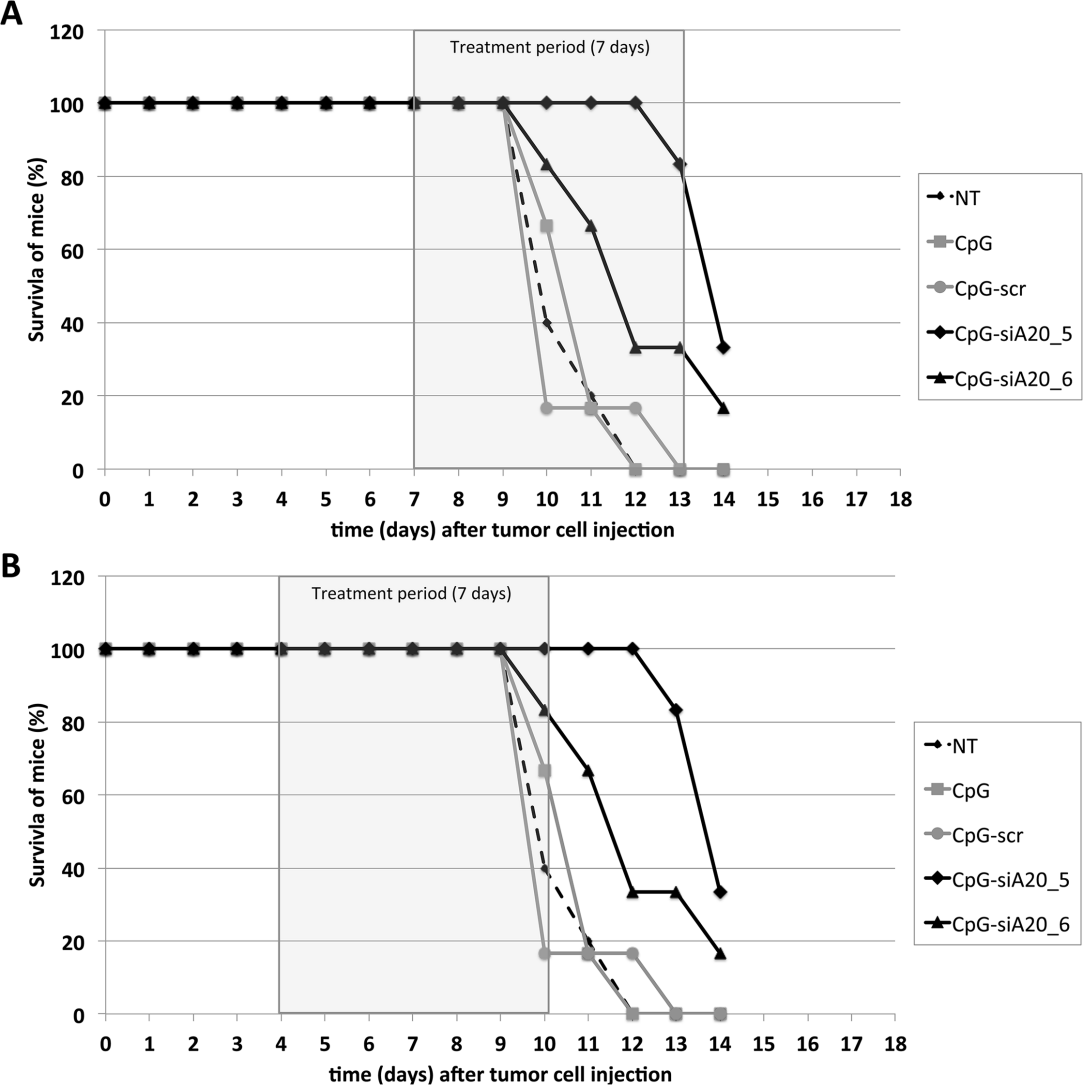
Tumor growing curves of B16 melanoma after CpG/ CpG-construct treatment. Subcutaneous injection of 1x10^6 B16 melanoma cells at day 0 followed by repetitive treatment (daily) with CpG/ CpG-scrambled RNA/ CpG-siRNA A20 for 7 days. Treatment with 1 nmol CpG/ CpG-constructs started 7 days after B16 cells injection **(A)**, started on day 4 **(B)** and with 2 nmol CpG/ CpG-constructs starting at day 4 **(C)**. Shown are mean tumor diameters in mm for six mice per group. **(D)** Statistical dot plot showing tumor diameters in the final phase of all three presented treatments (day 11 after tumor inoculation); n.s. indicates no significant difference, * indicates p<0.05, *** indicates p<0.001 (compared to CpG treated cells, multiple t-test).

**Fig 9:**
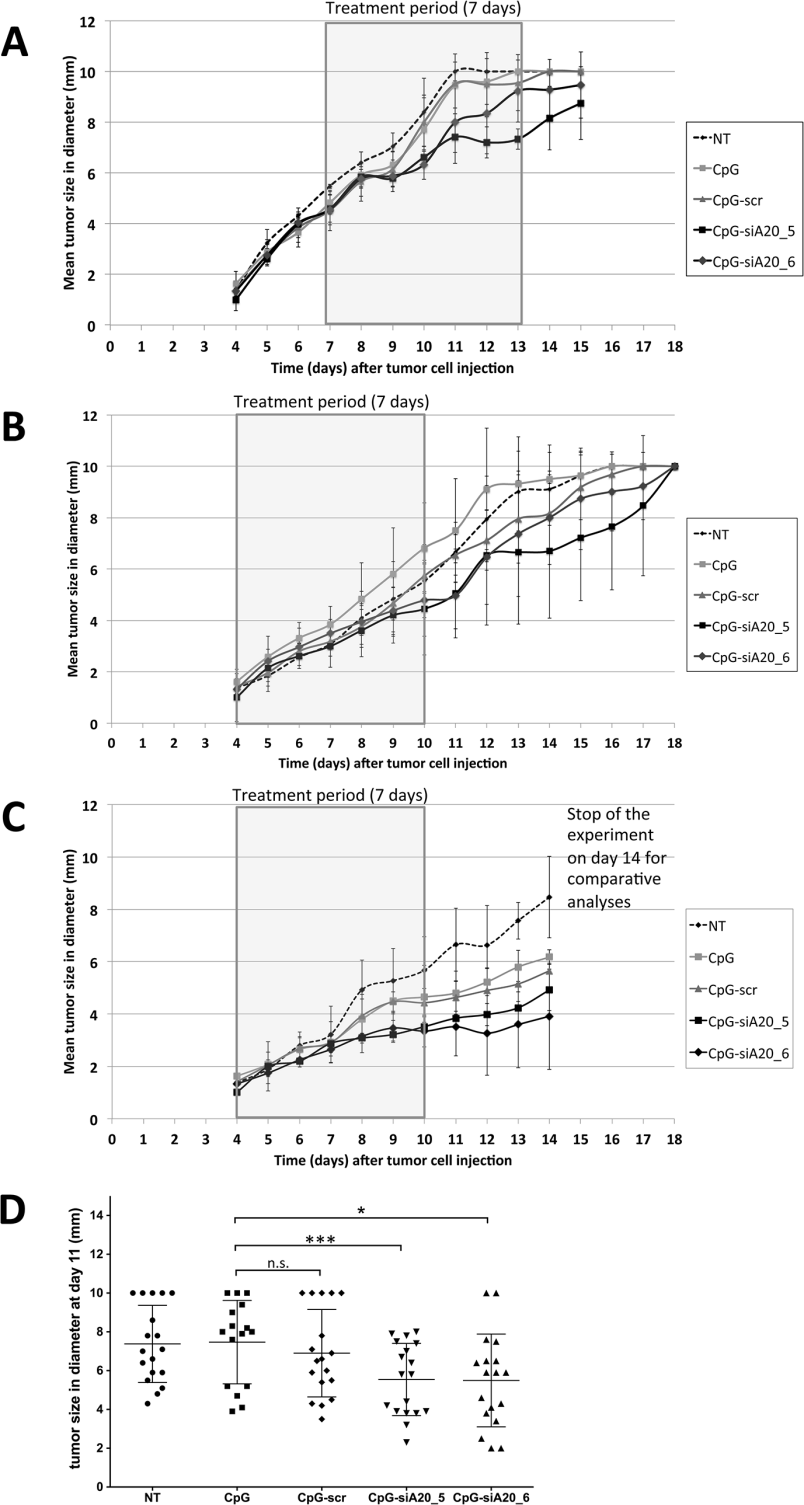
Survival curves of B16 melanoma-bearing mice after CpG/ CpG-construct treatment. Injection of 1x10^6 B16 melanoma cells s.c., repetitive treatment (daily) with CpG/ CpG-scrambled RNA/ CpG-siRNA A20. **(A)** Treatment started on day 7 until end of experiment on day 14 (tumor diameter ≥10 mm). **(B)** Treatment started on day 4 until day 10. Shown is percentage of surviving mice till the end of the experiment (tumor diameter ≥10 mm).

B16 melanoma cells developed aggressive tumors, which invariably killed the animals in the absence of treatment. The stimulation and activation of the innate and adaptive immune response in response to application of CpG or CpG-siRNA constructs resulted in less aggressive growth of B16 melanoma tumors. In the first set of experiments, administration of ODN-constructs was started 7 days after B16 cells had been inoculated. At this time point the tumors had reached approximately 5 mm in diameter. With this treatment schedule a slight inhibition of tumor growth could be achieved in mice injected with CpG-siA20 constructs (CpG-siA20_5, CpG-siA20_6). Mice from these two groups survived 3 to 4 days longer than the control animals until the tumor reached the limiting size (Fig 8A). Next, we modified the ODNs administration schedule: the oligonucleotides were injected earlier, 4 days after inoculation of the tumors. This modification further improved the survival of the mice, which received siA20-specific conjugates. Now tumor growth was markedly inhibited (S3 Fig) and in one group, CpG-siA20_5, half of the animals survived until day 18 on which they were sacrifice due to the tumor diameter approaching 10 mm (Fig 8B). The analysis of tumor growth curves at different treatment conditions revealed that the average tumor diameter remained smaller in animals treated with CpG-siA20 compared to PBS or CpG/CpG-siSCR(Fig 9). The most prominent effects were observed after doubling the dose of injected conjugates (Fig 9C). This trend failed to reach statistical significance when experiments were analyzed separately. However, integrated results obtained from all three presented experiments at the final phase of the treatment, indicated significantly smaller tumor diameter in A20-knockdown groups compared to CpG alone. In contrast, the scrambled CpG-siSCR conjugate and CpG treated tumors progressed with similar speed (Fig 9D).

**Fig 10:**
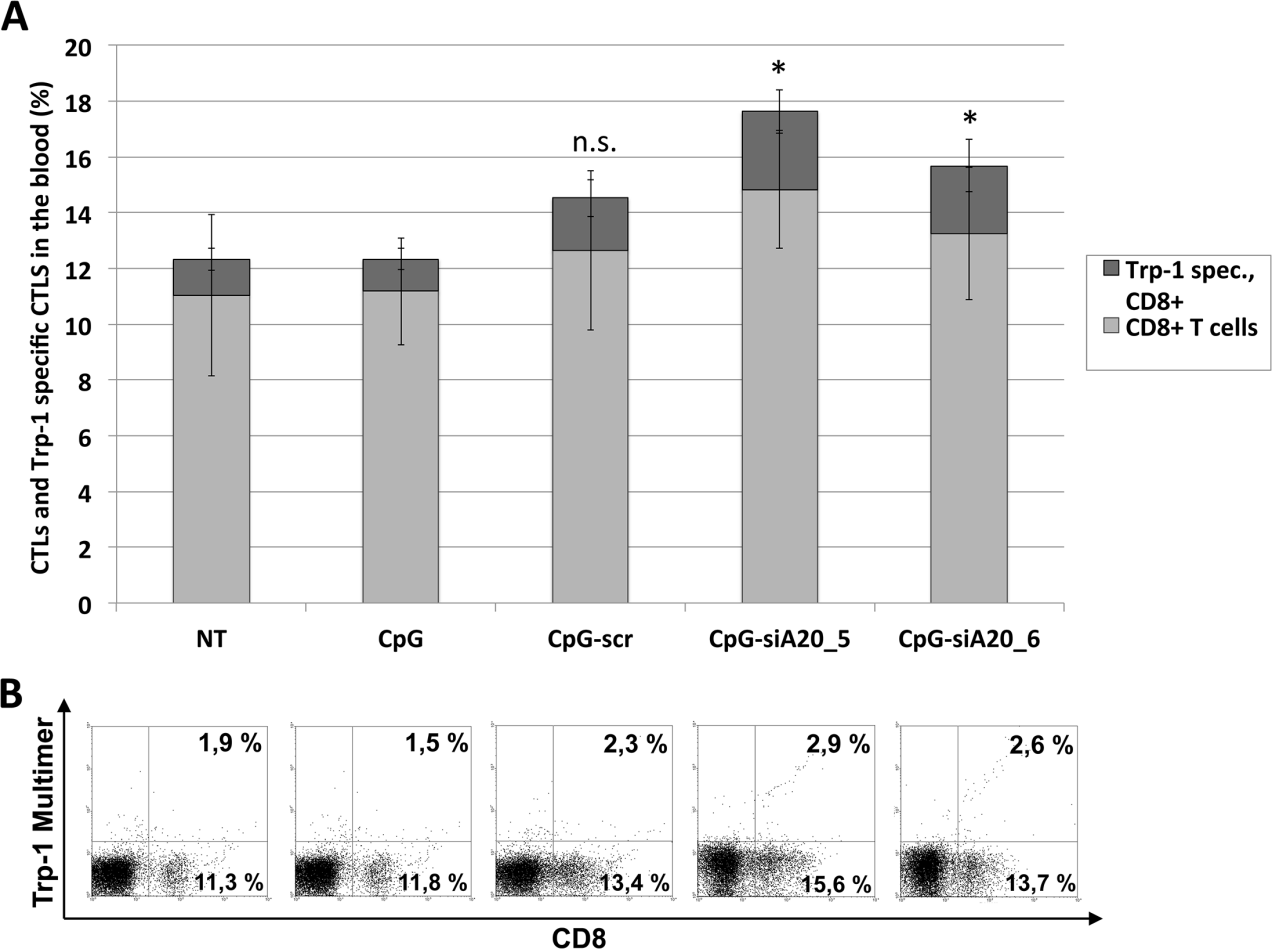
Tumor-specific CTLs in blood of B16 melanoma-bearing mice after CpG/ CpG-construct treatment. Injection of 1x10^6 B16 melanoma cells s.c. followed by repetitive daily treatment with 1 nmol CpG/ CpG-scrambled RNA/ CpG-siRNA A20 for 7 days started on day 4; blood collected for cellular analyses on day 10. Tumor-specific CD8+ T cells detected by Trp-1 multimer staining. **(A)** Mean percentage of total and Trp-1 specific CTLs (6 mice/group); n.s. indicates not significant, *indicates p<0.05 compared to CpG. Shown are results from one representative experiment. **(B)** Dot plots showing percentage of CTLs and Trp-1 specific CTLs in peripheral blood lymphocytes (one representative animal per group).

**Fig 11:**
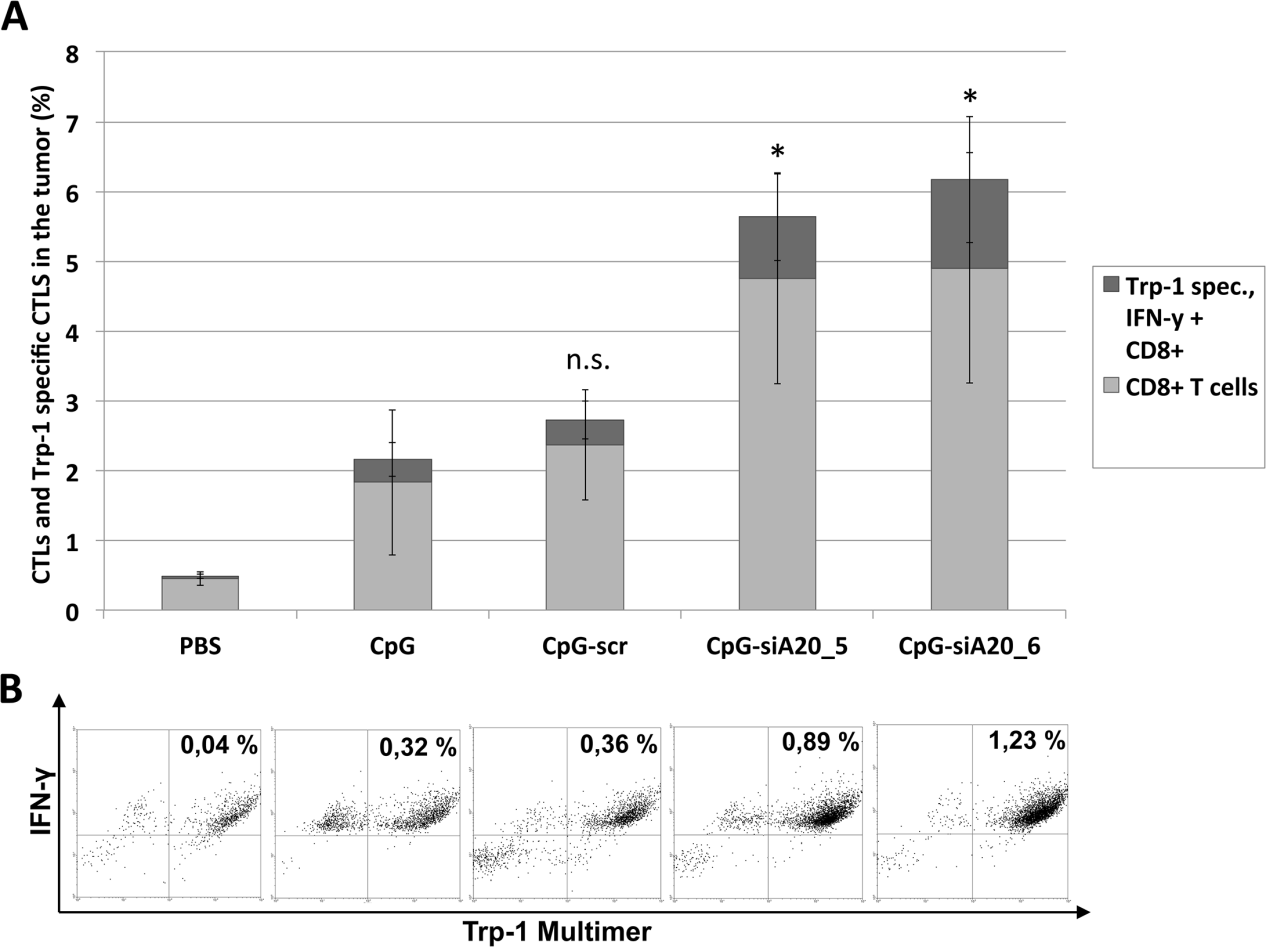
Tumor-specific CTLs inside B16 melanoma tumors after CpG/ CpG-construct treatment. Injection of 1x10^6 B16 melanoma cells s.c. followed by repetitive daily treatment with 2 nmol CpG/ CpG-scrambled RNA/ CpG-siRNA A20 for 7 days started on day 4; for all animals tumor tissue samples collected and analyzed on day 14. Detection of Trp-1 tumor-specific CD8+ T cells by multimer staining. Shown are results from one representative experiment. **(A)** Mean percentage of total and Trp-1 specific activated (IFN-γ positive) CTLs (6 mice/group); n.s. indicates not significant, *indicates p<0.05 compared to CpG. **(B)** Dot plots showing frequency of activated (IFN-γ positive) Trp-1 specific CTLs in tumor-derived lymphocyte fraction (one representative animal per group).

We next investigated whether and how CpG or CpG-siA20 oligonucleotides induced any tumor specific immune response. Circulating immune cells isolated from treated or untreated tumor-bearing mice were analyzed before tumor inoculation and 6 days after beginning of treatment. They were stained with FITC-labeled multimers presenting peptide derived from tumor antigen Trp1, which is expressed by B16 cells, in the context of MHC class I haplotype H2^b^. This complex binds exclusively to Trp1-specific T cell receptors (TCRs). As shown in Fig 10, the percentage of CD8-positive cells was comparable regardless of the applied treatment. In contrast the frequency of CD8 positive cells co-stained with the Trp1-pentamer increased slightly but significantly in animals treated with CpG-siA20 constructs. Next, we focused on immune cells repertoire within the tumor tissue. In order to avoid the potential influence of the tumor growth period on its cellular composition the tumors from all animal groups were acquired simultaneously 10 days after treatment start and before they reached critical size. Unlike in peripheral blood, the frequency of CD8+ cells within CpG-siA20 treated tumors was markedly higher than in controls (Fig 11A). Moreover, the significant fraction of CpG-siA20 tumor lymphocytes was positive for both Trp-1-specific TCR and activation marker intracellular IFNγ (Fig 11B). The rate of tumor-specific CD8+ cells correlated with retardation of tumor growth indicating the efficient induction of a tumor-specific CTL response upon the combined TLR stimulation and A20 attenuation.

**Fig 12:**
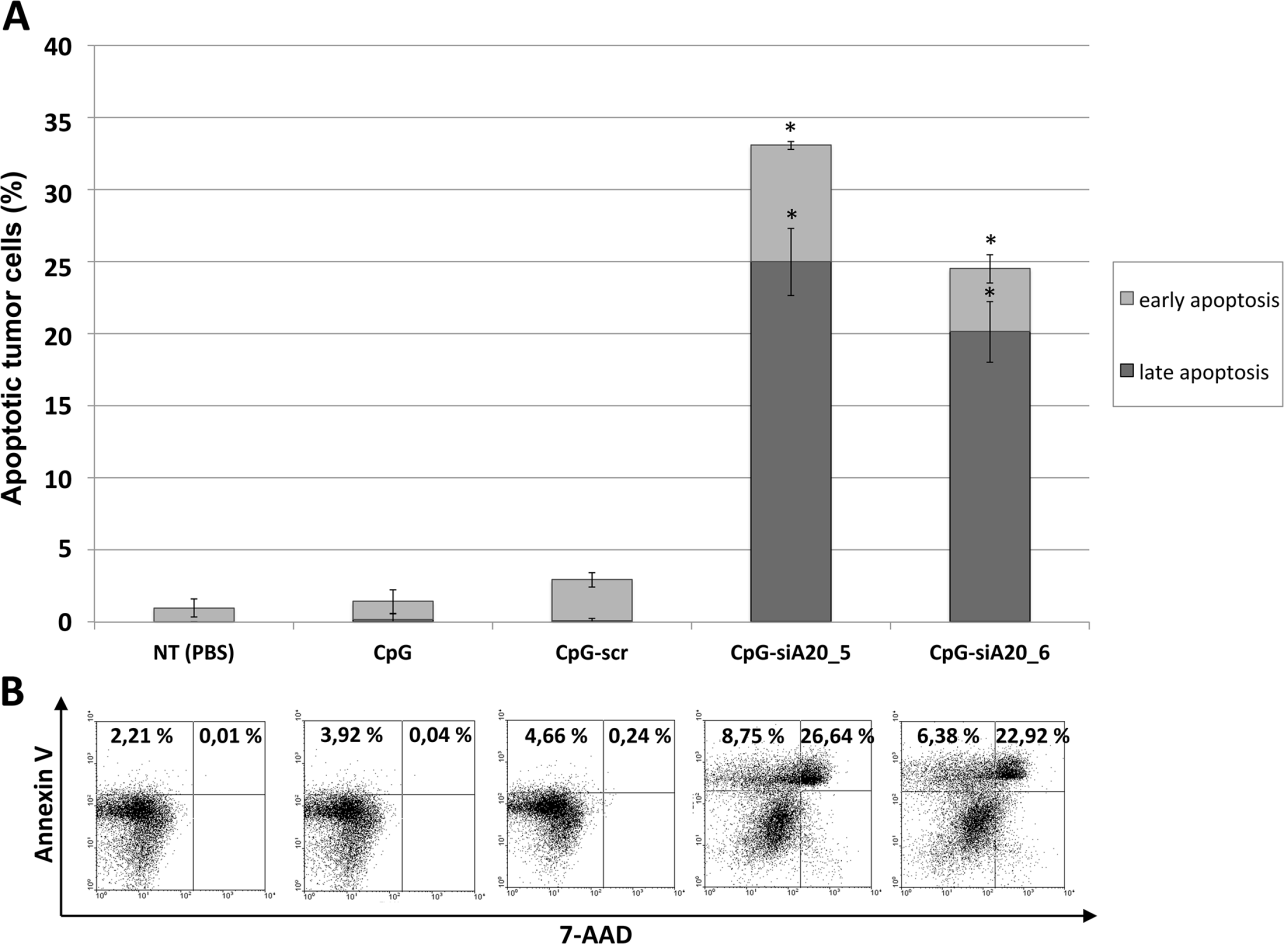
B16 tumor cell apoptosis after CpG/ CpG-construct treatment. Injection of 1x10^6 B16 melanoma cells s.c. followed by repetitive treatment (day 4–10) with CpG/ CpG-scrambled RNA/ CpG-siRNA A20. Apoptosis measured in tumors isolated at the end of experiment. **(A)** Shown are mean percentage of early (Annexin V positive) and late (7-AAD and Annexin V positive) apoptotic cells (6 mice/ group); n.s. indicates not significant, *indicates p<0.05 compared to CpG. **(B)** Dot plots showing percentage of apoptotic cells for one representative animal per group.

Since activation of the cytotoxic arm of the immune system should result in killing of infected or transformed cells, we checked the tumors for the presence of dead or dying cells using Annexin V/7-AAD staining assay. While CpG alone and CpG-scr construct did not cause significant apoptosis within the tumor, the percentage of dead cells in A20-depleted groups was substantially elevated, reaching up to 25% of all B16 cells (Fig 12).

## Discussion

Tolerance mechanisms, which are essential for preventing autoimmune responses, represent the main obstacle in developing efficient antitumor vaccines, which require activation of the immune system against tumor associated self-antigens (TAAs). The central role in switching the immune status between tolerant and activated states is played by DCs. Depending on their maturation state and the cytokines they express DCs may either create immune suppressive microenvironment or support the expansion of tumor-associated antigen specific T cells. The maturation of DCs required for effective immune response can be accelerated by providing stimuli such as inflammatory cytokines or cross-linking of costimulatory receptors (CD40, GITR-L, OX40L). It has been demonstrated that activated through engagement of their TLRs DCs are capable of switching from a tolerogenic mode to induce TAA-specific cytotoxic T cells instead. Triggering TLRs activates multiple transcription factors, which ultimately induce the expression of cytokines and surface molecules characteristic for mature fully functional DCs [21], [22], [23]. However, since the excessive activation is potentially harmful it needs to be tightly controlled. The TNFAIP3 gene encoding the A20 protein has been recently identified as an essential factor negatively regulating the maturation of DCs, their production of inflammatory cytokines and general stimulatory properties. Selective inhibition of A20 in mouse DCs *ex vivo* results in induction or augmented expression of costimulatory molecules and the pro-inflammatory cytokines IL-6 and TNF-α, which eventually lead to significantly reduced activity of Tregs and enhanced priming and effector phases of immune response against tumors. Silencing of A20 in human monocyte-derived DCs, however, revealed that this inhibition was not sufficient for the enhanced DC maturation, cytokine production or stimulation of the adaptive immune responses but required additional TLR3 stimulation with poly(I:C) [19].

In this study, we therefore adopted a strategy established by Kortylewski and coworkers [15], and synthesized a molecule that combines the immunostimulatory properties of a TLR9 ligand with A20 inhibition. In addition, the molecule preferentially targets DCs by virtue of its TLR9-binding properties. Treatment of bone marrow derived DCs or a DC cell line with CpG-siA20 conjugates led to rapid construct internalization and A20 downregulation. This was followed by elevated NF-κB signaling and cytokine secretion, which were superior compared to CpG alone or to CpG fused with irrelevant siRNA. When analyzed on the protein level, A20 was upregulated initially in all samples treated with CpG or CpG-siRNA constructs. At a later time point, 20 hours after treatment, the amount of A20 protein diminished selectively in cells treated with CpG-siA20 constructs, however, already 4 hours later the A20 protein signal had returned to higher levels than those in control cells. In our opinion the reason for that phenomenon could be the fact that A20 is a NF-κB target itself. Hence, the augmented activity of NF-κB due to initial A20 depletion ultimately leads to a rebound with increased A20 levels along with other NF-κB targets like IκBα, IL-6 or TNF-α.

The CpG-siA20 construct can be applied *in vivo* without the need of any additional transfer or vector systems, a major advantage compared with other approaches aiming A20-depletion. Moreover, no major toxicity was detected upon systemic administration of CpG-siA20 into healthy mice. The levels of proinflammatory cytokines in serum increased in all animals, who received CpG or CpG fused to siRNA. However, A20 specific siRNA constructs increased the concentration of IL-6, TNF-α and IFN-γ much more than CpG alone or its fusion with irrelevant siRNA. Peritumoral administration of CpG and CpG conjugates caused a similar shift of the cytokine profile in peripheral blood but the magnitude of the effects was dependent on the treatment schedule. Regardless of the stage of tumor growth, the injection of conjugates increased serum concentrations of IL-6, TNF-α, IFN-γ and IL-12p70. The elevation of IL-12 may be particularly important. It has been shown recently in human monocyte-derived DCs that the immunostimulatory effect of A20 depletion, measured as the capacity to induce a Th1 response, is critically dependent on the secretion of this cytokine by DCs. This is in agreement with the well-known fact that IL-12 promotes activation of NK, NKT, Th1 and Tc1 cells [24], [25], [26]. Interestingly, IL-10, an immunosuppressive cytokine known to be expressed by DCs was elevated in all treated groups regardless of the targeting of A20. However, only in mice treated with CpG-siA20 conjugates the rise of soluble inflammatory factors in the serum was accompanied by a dramatic increase of the percentage of tumor-antigen-specific CD8+ T cells. Although the tumor progression could not be stopped in these animals, simultaneous stimulation of TLR9 and A20 knockdown resulted in its substantial delay. A strong induction of cell death within these tumors clearly correlated with slower growth. Why such a strong activation of the immune system did not lead to tumor eradication remains to be addressed. First, B16 is among the most aggressive tumor types, refractory to most treatment approaches [27],[28]. And then we could demonstrate, the outcome differed depending on the size of tumor at treatment initiation. We assume the difference between A20-inhibited and control animals could have been magnified by further reducing the interval between tumor inoculation and injection of the conjugates. However, we preferred to study a system where the tumors were well established before treatment opting for treatment rather than tumor prevention schedule. On the other hand, there are multiple parameters that might be changed to optimize the activity of the conjugates. The repertoire of synthetic CpG oligonucleotides differing in their immunostimulatory properties and cell specificity is constantly expanding and some types are already submitted to the pre-/clinical trials [29], [30], [31]. The siRNA component of the conjugate is another candidate for optimization. As demonstrated by multiple studies, much higher silencing activities could be obtained by increasing the length of siRNA oligonucleotides [32]. Even better efficiency may be obtained when the gene targeting sequence is presented in the context of the naturally occurring miRNA backbones [33]. Moreover, increasing the serum stability and reducing the negative charge of the conjugates by chemical modifications might result in more efficient and sustained A20 knockdown [34]. Another attractive option to improve the presented strategy would be simultaneous targeting of A20 in multiple immune compartments. A recent report of Giordano *et al*. [35] demonstrated significantly increased sensitivity to antigen stimulation and elevated expression of IL-2 and IFN-γ in CD8+ T cells in which A20 was attenuated. Aptamers capable of binding and delivering siRNA cargo specifically to T cells have been described recently and could represent an ideal tool for that purpose [36], [37].

Taken together, our results indicate that simultaneous activation of TLR9 and attenuation of A20 expression in DCs using *in vivo* deliverable CpG-siRNA conjugate is capable of inducing a strong and specific immune response against an established tumor. This approach represents an attractive alternative to current therapeutic trials employing TLR agonists alone and/or manipulating DCs *ex vivo*.

## Supporting Information

S1 Fig CpG-siA20 oligonucleotide design and proposed mechanism of action. The CpG-siA20 construct consists of a single strand DNA oligonucleotide binding endosomal TLR9 *e*.*g*. in DCs and a double strand siRNA against A20. Construct uptake leads to enhanced NF-κB activation resulting in augmented stimulatory functions of antigen presenting cells (DCs, macrophages) followed by expansion of antigen-specific cytotoxic T lymphocytes.
(TIF)

S2 Fig Long-term BMDC culture. Surface marker CD11c, B220, CD80, CD86, and MHC class II. **(A)** From bone-marrow preparation (day 0) until day 26. **(B)** From day 0 until day 300. Shown are surface marker positive cells in %, measured by FACS (see methods). **(C)** Confocal images of BMDCs from day 14, nucleus stained with Nuc Blue Live Cell Stain (ex 405), cellular membrane stained with Oregon Green 488 DHPE (ex 448), aquisition using Olympus Fluoview FV1000 LSM microscope, FV1200 ASW system software.
(TIF)

S3 Fig Murine melanoma tumor model. B16 melanoma cells (1x10^6) were injected subcutaneously in the right flank of 8–10 week old female C57BL/6 mice. The pictures are showing one representative tumor on day 10 after B16 melanoma cell injection. **(A)** Three repetitive treatments with CpG-siA20 construct. **(B)** Three repetitive treatments with CpG. **(C)** PBS treatment.
(TIF)

S1 Materials and Methods RT-PCR Primer Sequences. (DOC)
