# Supplementary figures and images for: In Vivo Silencing of A20 via TLR9-Mediated Targeted SiRNA Delivery Potentiates Antitumor Immune Response
Source: PLoS One. 2015 Sep 1;10(9):e0135444. doi: 10.1371/journal.pone.0135444 (PMC4556692; doi:10.1371/journal.pone.0135444)

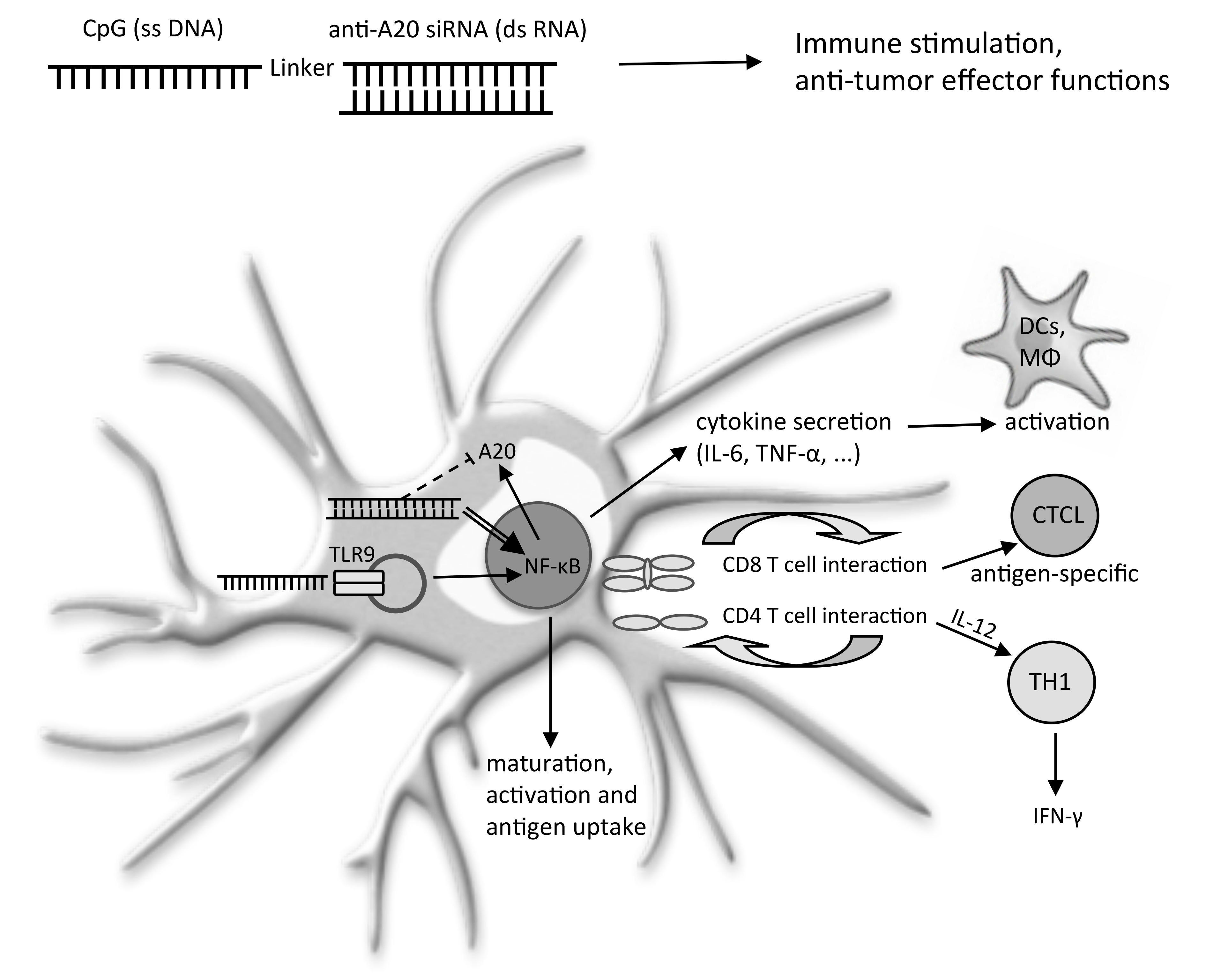

Supplement: S1 Fig — The CpG-siA20 construct consists of a single strand DNA oligonucleotide binding endosomal TLR9 e.g. in DCs and a double strand siRNA against A20. Construct uptake leads to enhanced NF-κB activation resulting in augmented stimulatory functions of antigen presenting cells (DCs, macrophages) followed by expansion of antigen-specific cytotoxic T lymphocytes. (TIF) [file pone.0135444.s001.tif]

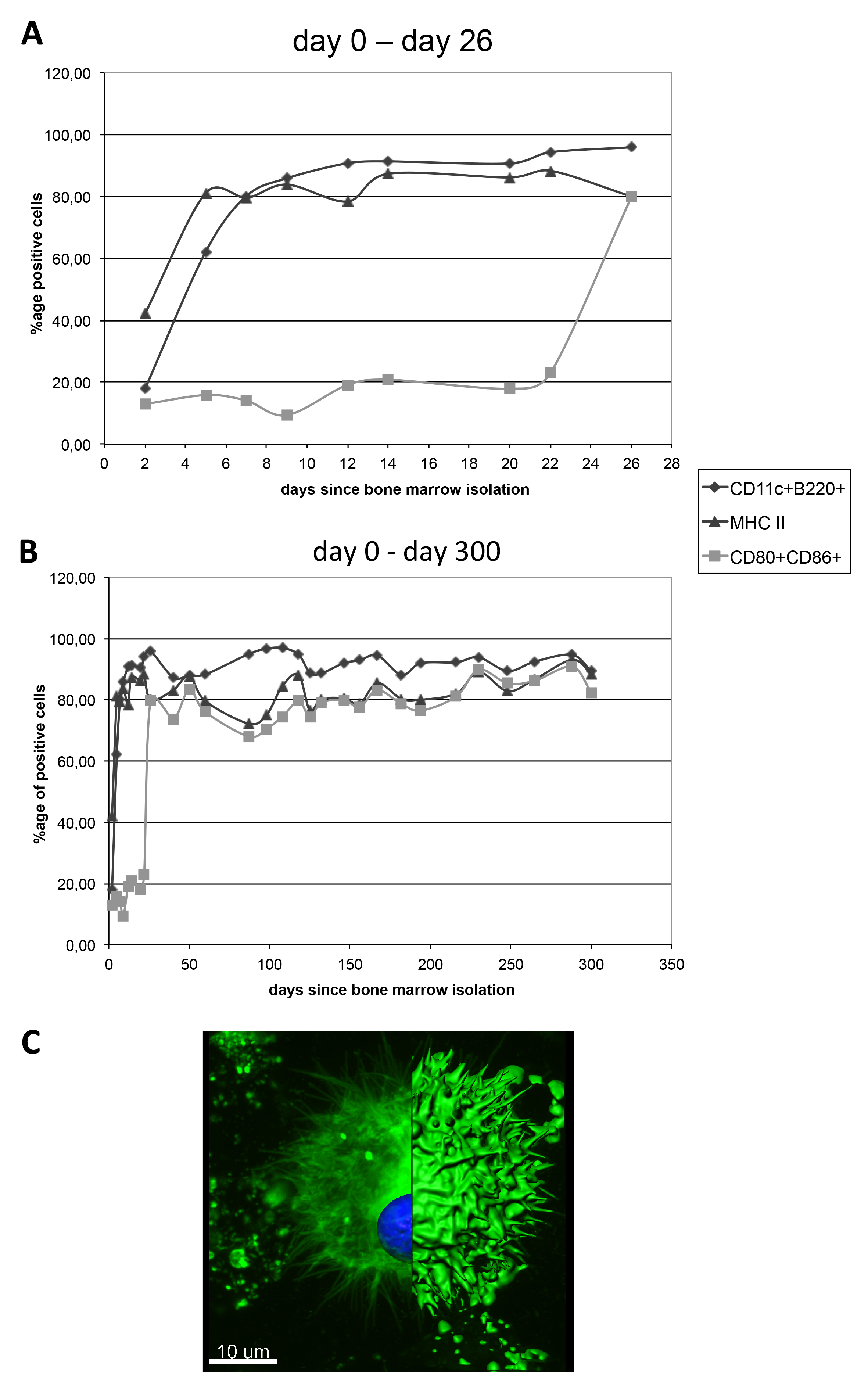

Supplement: S2 Fig — Surface marker CD11c, B220, CD80, CD86, and MHC class II. (A) From bone-marrow preparation (day 0) until day 26. (B) From day 0 until day 300. Shown are surface marker positive cells in %, measured by FACS (see methods). (C) Confocal images of BMDCs from day 14, nucleus stained with Nuc Blue Live Cell Stain (ex 405), cellular membrane stained with Oregon Green 488 DHPE (ex 448), aquisition using Olympus Fluoview FV1000 LSM microscope, FV1200 ASW system software. (TIF) [file pone.0135444.s002.tif]

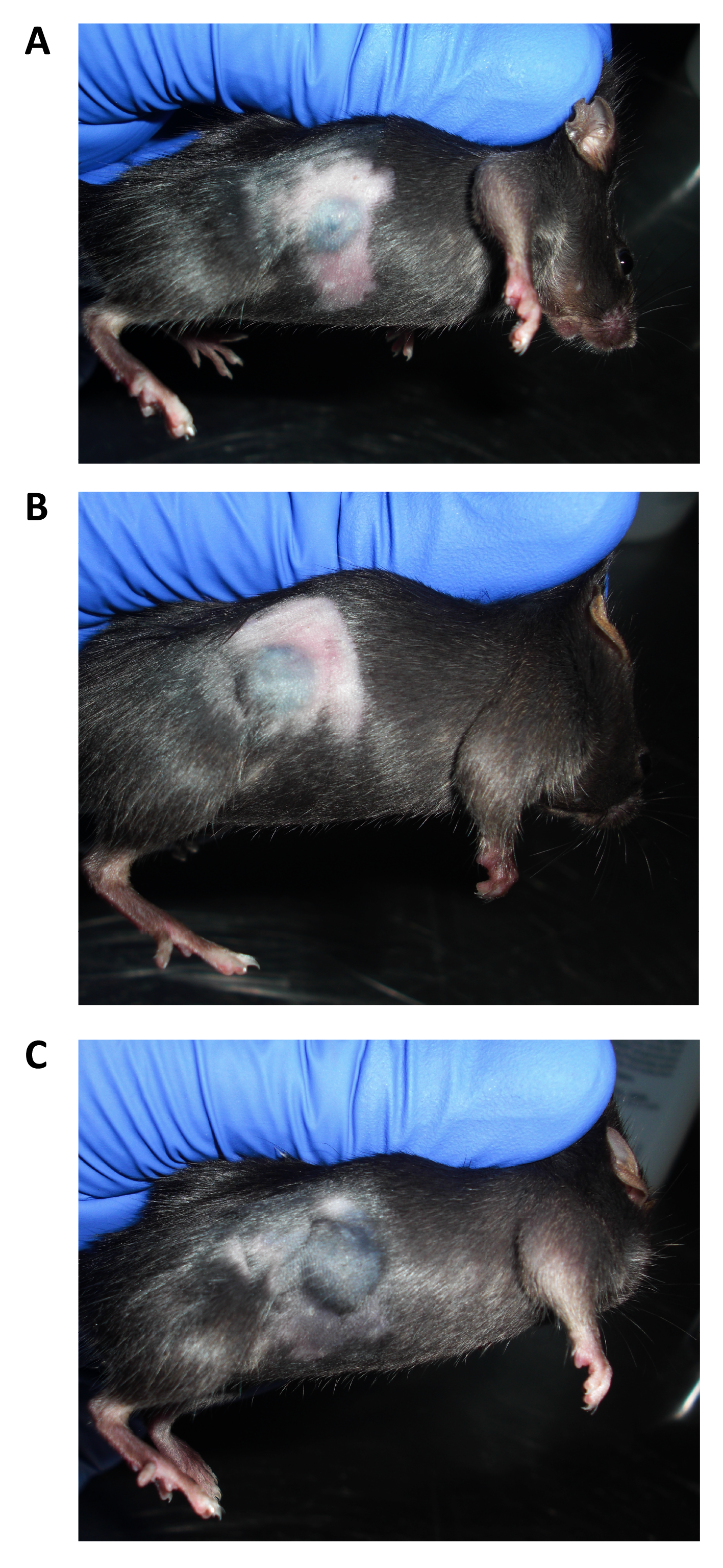

Supplement: S3 Fig — B16 melanoma cells (1x10^6) were injected subcutaneously in the right flank of 8–10 week old female C57BL/6 mice. The pictures are showing one representative tumor on day 10 after B16 melanoma cell injection. (A) Three repetitive treatments with CpG-siA20 construct. (B) Three repetitive treatments with CpG. (C) PBS treatment. (TIF) [file pone.0135444.s003.tif]
