# Supplementary material for: In Vivo Silencing of A20 via TLR9-Mediated Targeted SiRNA Delivery Potentiates Antitumor Immune Response
Source: PLoS One. 2015 Sep 1;10(9):e0135444. doi: 10.1371/journal.pone.0135444 (PMC4556692; doi:10.1371/journal.pone.0135444)
Supplement: S1 Materials and Methods — (DOC) [file pone.0135444.s004.doc]

S4. Materials and Methods

RT-PCR Primer Sequences:

| **Name** | **Length in bp** | **Sequence** |  |
| --- | --- | --- | --- |
| mB2MGf | 20 | 5`- AAgTATACTCACgCCACCCA | forward |
| mB2MGr | 20 | 5`- AAgACCAgTCCTTgCTgAAg | reverse |
| mTNFAIP3-f-3 | 20 | 5`- CACTCggAAgCACCATgTTTg | forward |
| mTNFAIP3-r-3 | 20 | 5`- gCTTAggTgCTggCACTCCAT | reverse |
| mIL6-f-44 | 20 | 5`- TgggACTgATgCTggTgACA | forward |
| mIL6-r-254 | 22 | 5`- TCTgCAAgTgCATCATCgTTgT | reverse |
| mTNF-a-f-77 | 20 | 5`- ACTCCAggCggTgCCTATgT | forward |
| mTNF-a-r-326 | 20 | 5`- CTCAgCCACTCCAgCTgCTC | reverse |
| IkBa 2-for | 20 | 5`- TggAgCACTTggTgACTTTg | forward |
| IkBa 2-rev | 20 | 5`- TggAgATTTTCCAgggTCAg | reverse |
